# The St. John's Hospital Scandal

**Published:** 1888-03-17

**Authors:** 


### THE ST. JOHN'S HOSPITAL SCANDAL.

IT is with deep pain and regret that we feel it our duty, for the first time, to call attention to discreditable facts in connexion with the management of a particular hospital. Charges have recently been made against the responsible managers of *St. John's Hospital for Diseases of the Skin*, Leicester Square, of the very gravest nature. Those charges, taken in conjunction with previous scandals, made it absolutely imperative upon the management to institute an inquiry of the most searching character, and to satisfy the public that they were either true or false. If such an inquiry had proved the charges true, then an immediate change of administration was called for; if, on the contrary, their falsehood had been demonstrated, legal action should have been commenced against the traducers, with a view to their prompt and effectual punishment.

What course, however, did the Governors of the St. John's Hospital think it proper to take? At first, all inquiry of every kind was absolutely refused. When, in the face of repeated charges, such a line of conduct could no longer be persisted in, a meeting of the Governors was called; but there is no evidence whatever to show that those who attended the meeting and professed themselves satisfied were *all bona fide* governors of the institution. Again, we have to record the significant fact that the Duke of Northumberland, the President of the hospital, has felt it incumbent upon him to cease to hold that position, and to withdraw his countenance and support.

In spite of the retirement of the President, and of the reiterated charges published in *Truth*, whose editor deserves the thanks of the public and of all honest hospital officials and managers for his straightforward conduct in this matter, no steps have yet been taken either to clear the accused or to punish the accuser. It is to be wished that not only *The Star* but every other metropolitan paper would enlighten the public as to the reasons why they should not support St. John's Hospital under present circumstance.

What can the public understand in such a case as this? There is only one conclusion possible: and that, we do not hesitate to say, is not such as should satisfy the Governors. For our part we have but one duty to perform, and that is to state as plainly as words will allow that the interests of hospitals and the public alike demand that all support should be at once withdrawn from this institution, and that such withdrawal should be permanent unless there shall be a complete and open inquiry by independent parties which shall clear the management; or such an entire change in the administration as shall make it entirely new. Hospital managers of every rank and degree should not merely be above suspicion, but should always be prepared to produce the completest evidence not only of their good intentions, but of their spotless integrity.

